# Incidence rate and histology of appendiceal neoplasms in complicated versus uncomplicated appendicitis: A meta-analysis and systematic review

**DOI:** 10.1007/s00423-023-03164-0

**Published:** 2023-11-09

**Authors:** Paola Solis-Pazmino, Kimberly Oka, Kristina La, Orly Termeie, Luis A. Figueroa, Eduardo Pilatuna, Daniel Solis-Pazmino, Mary Pat Harnegie, Jason Cohen, Moshe Barnajian, Yosef Nasseri

**Affiliations:** 1 Surgery Group Los Angeles, Los Angeles, CA USA; 2 Surgery Department, Santa Casa de Porto Alegre, Porto Alegre, RS Brazil; 3 Knowledge and Evaluation Research Unit, Mayo Clinic, Rochester, MN 55905 USA; 4 CaTaLiNA- Cancer de Tiroides en Latino América, Quito, Ecuador; 5 Facultad de Ciencias Médicas, Universidad Central del Ecuador, Quito, Ecuador; 6 Universidad San Francisco de Quito, Quito, Ecuador; 7 Facultad de Odontología, Universidad Central del Ecuador, Quito, Ecuador; 8 Cleveland Clinic, Cleveland, OH USA; 9 Cedars-Sinai Medical Center, Los Angeles, CA USA

**Keywords:** Appendicitis, Complicated, Uncomplicated, Abscess, Phlegmon, Perforation, Neoplasm

## Abstract

**Introduction:**

Studies evaluating the rate and histology of appendiceal neoplasms between complicated and uncomplicated appendicitis include a small number of patients. Therefore, we sought a meta-analysis and systematic review comparing the rates and types of appendiceal neoplasm between complicated and uncomplicated appendicitis.

**Methods:**

We included articles published from the time of inception of the datasets to September 30, 2022. The electronic databases included English publications in Ovid MEDLINE In-Process & Other Non-Indexed Citations, Ovid MEDLINE, Ovid EMBASE, and Scopus.

**Results:**

A total of 4962 patients with appendicitis enrolled in 4 comparative studies were included. The mean age was 43.55 years (16- 94), and half were male (51%). Based on intra-operative findings, 1394 (38%) had complicated appendicitis, and 3558 (62%) had uncomplicated appendicitis. The overall incidence rate of neoplasm was 1.98%. No significant difference was found in the incidence rate of appendiceal neoplasm between complicated (3.29%) and uncomplicated (1.49%) appendicitis (OR 0.44, 95% CI 0.16- 1.23; p < 0.087; I2 = 54.9%). The most common appendiceal neoplasms were Neuroendocrine Tumors (NET) (49.21%), Nonmucinous Adenocarcinoma (24.24%), Mixed Adeno-Neuroendocrine Tumor (MANEC) (11.40%), Mucinous Adenocarcinoma (4.44%). There was a significant difference between complicated and uncomplicated appendicitis in rates of adenocarcinoma (50% vs. 13%), NET (31% vs. 74%), MANEC (19% vs. 13%) (P < 0.001).

**Conclusion:**

While there was no significant difference in the overall neoplasm rate between complicated and uncomplicated appendicitis, the NET rate was significantly higher in uncomplicated appendicitis. In comparison, the Adenocarcinoma rate was considerably higher in Complicated appendicitis. These findings emphasize the importance of evaluating risk factors for neoplasm when considering appendectomy in patients with appendicitis.

**Supplementary information:**

The online version contains supplementary material available at 10.1007/s00423-023-03164-0.

## Introduction

Appendicitis is one of the most common surgical pathologies. Despite its commonality, the incidence of appendiceal neoplasms (APNs) associated with complicated versus uncomplicated appendicitis remains unclear. The several studies that have addressed this topic include a small number of patients. There is also a need for more data about types of appendiceal neoplasm associated with complicated and uncomplicated appendicitis. To obtain a broader understanding of appendiceal neoplasms in the setting of appendicitis, we conducted a meta-analysis and systematic review comparing the rates and types of appendiceal neoplasms between complicated and uncomplicated appendicitis.

## Methods

The study followed the PRISMA guidelines for systematic reviews and meta-analysis. The protocol was registered at PROSPERO (CRD42023398888).

Eligibility criteria included observational and experimental studies that compared the rates and/or types of appendiceal neoplasms between complicated and uncomplicated cases of appendicitis. Complicated appendicitis (CAP) had patients with abscess, phlegmon, and/or perforated appendicitis, as determined during surgery. The primary outcome was the incidence of appendiceal neoplasms (APN), with secondary outcomes being the different types of APN: low-grade mucinous neoplasm (LGMN), high-grade mucinous neoplasm (HGMN), adenocarcinoma, adenosquamous carcinoma, neuroendocrine tumors, mixed adeno-neuroendocrine carcinoma (MANEC), pseudomyxoma, lymphoma, and adenoma or serrated lesions.

Studies that focused on preoperative findings of complicated or uncomplicated appendicitis were excluded, as well as conference abstracts, literature reviews, and editorials.

### Data sources and searches

An extensive search was performed across multiple databases from their inception until September 2022 to conduct a thorough and systematic analysis of the available evidence. The selected databases included sources such as Ovid MEDLINE(R) and Epub Ahead of Print, CINAHL, Ovid EMBASE, Ovid Cochrane Central Register of Controlled Trials, Ovid Cochrane Database of Systematic Reviews, Scopus, and Web of Science. The Web of Science search encompassed the Science Citation Index (SCI), Conference Proceedings Citation Index (CPCI), and BIOSIS Citation Index (BCI) to ensure comprehensive coverage.

The principal investigator meticulously curated a search strategy in collaboration with an expert librarian. This involved identifying relevant controlled vocabulary and keywords specific to the research domain. The search strategy was designed to be rigorous and aimed at retrieving all pertinent studies, leaving no stone unturned in pursuing a comprehensive evidence base.

### Study selection

The search records were processed using the Covidence systematic review software from Veritas Health Innovation in Melbourne, Australia. The review process, including title and abstract screening, full-text screening, and data extraction, was conducted by pairs of independent reviewers (P.S-P., K.O., K.L, E.P, L.F). Pilot tests were conducted before each stage to ensure an accurate understanding of quality criteria. In case of disagreement, the senior author (Y. N.) provided a resolution. The agreement in full-text screening was evaluated using Cohen's kappa. (k = 0.72).

### Data collection

The extraction of data involved several critical elements of the studies being reviewed. These included general characteristics such as the first author, publication date, country of analysis, study design, and data collection period. The setting of the study, whether it was conducted at a single center or multiple centers, was also recorded. In addition, the patient’s preoperative characteristics were extracted, including their age and sex. The primary outcome of interest was the incidence of appendiceal neoplasm (APN), while the secondary outcomes were the types of appendiceal neoplasms.

### Risk of bias assessment

The study quality was evaluated by five independent reviewers (P.S-P., K.O., K.L, E.P, L.F), with disagreements resolved by consensus by two reviewers (Y.N., P.S-P.). The risk of bias in cohort studies was assessed using the CLARITY tool, which evaluated eight aspects of the study, including the selection of exposed and unexposed cohorts, the confidence in exposure and outcome assessments, and the adequacy of follow-up and control for co-interventions. Each aspect was given a rating of "definitively yes," "probably yes," "probably no," or "definitively no." "Definitively yes" was considered low risk, "probably yes" and "probably no" was considered a moderate risk, and "definitively no" was considered high risk. The overall risk of bias was calculated based on the responses to each of the eight questions. Studies with two or more "high risk of bias" questions were considered a high overall risk, and those with at least one "high risk of bias" question were considered a moderate overall risk. Those with three or more "moderate risk of bias" questions were considered high overall risk. Studies with only "low risk of bias" questions were supposed to have a low overall risk. This approach has been used previously [1].

### Certainty in the body of evidence

The certainty of the evidence was evaluated using the Grading of Recommendations Assessment, Development, and Evaluation (GRADE) approach. This evaluation reflects the confidence level that the systematic review results are accurate. Two reviewers (P.S-P, L.F) individually assessed the quality of evidence, and any disagreements were resolved through consensus with the involvement of a third reviewer (Y.N). The quality of evidence for each treatment-comparison-outcome can be classified as very low, low, moderate, or high. The initial rating for randomized trials was considered high-quality, and observational studies were regarded as low-quality evidence. The final rating was adjusted using the GradePro GDT (Cochrane) tool, taking into account factors such as the risk of bias, inconsistency, indirectness, imprecision, and publication bias (which downgrade the rating), and large magnitude of effect, plausible confounding, and dose–response gradient (which upgrade the rating).

### Statistical analyses

The statistical analysis used an intention-to-treat approach for dichotomous outcomes, calculating each study's odds ratio (OR) and 95% confidence interval (CI). The 95% CI for continuous variables was calculated using a random-effects model with the restricted maximum likelihood (REML) method. RStudio, a software for R programming, was utilized to perform the analysis and create forest plots. Heterogeneity among studies was evaluated using the study variance estimate (tau squared). The I2 statistic was used to measure the proportion of variability in effect size estimates due to between-study heterogeneity. Medians were converted to means and ranges or interquartile ranges to standard deviations (SDs), and the means and SDs of each variable were combined using weighted mean and weighted SD [2].

## Results

### Search results

**Fig. 1:**
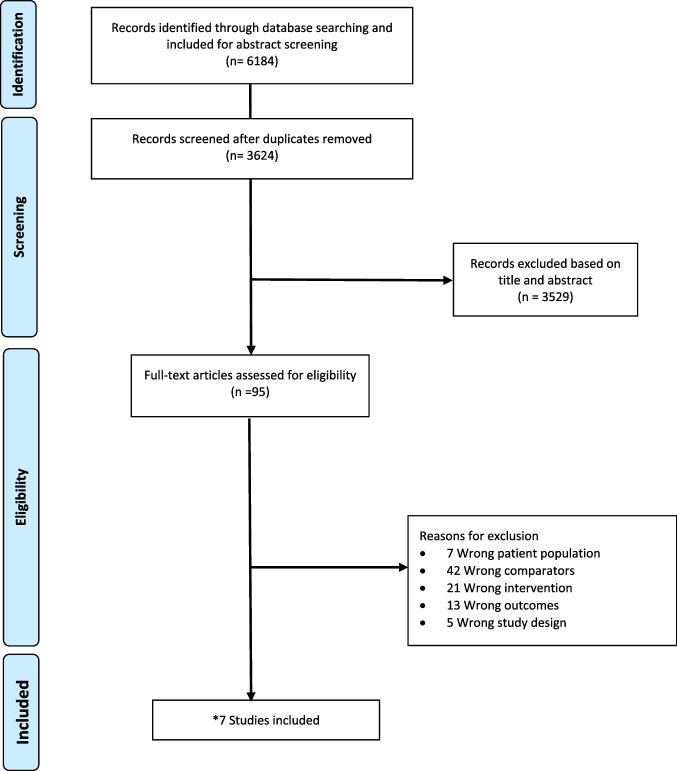
PRISMA flow diagram of the study selection process

After performing deduplication and thorough screening, we identified 6,184 studies from the initial literature search. Following a meticulous evaluation against the inclusion criteria, 4 studies were deemed eligible to analyze the primary outcome. Furthermore, 3 additional papers (n = 7) fulfilled the criteria and were included in the secondary outcome analysis (Fig. 1).

### Study characteristics

All seven were comparative cohort studies. Six retrospectives [3–8] and one prospective [9] study were published from 2017 to 2022. The risk of bias was low in 4 studies and 3 moderated. The studies were conducted in Europe (4 studies), the United States (2 studies), and Japan (1 study).

### Primary outcome

#### Risk of appendiceal neoplasm (APN)

**Fig. 2:**
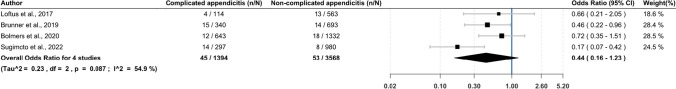
Primary outcomes

The incidence of APN was reported in 4 studies [3–7, 9] covering 4962 appendectomies (Table 1). The overall incidence of neoplasm in appendectomy specimens was 1.98%. The incidence of APN in CAP (n = 1394) was 3.29%, while the incidence of APN in UCAP (n = 3558) was 1.49%. The risk of having an APN was comparable between patients with CAP and UCAP, with an odds ratio of 0.44 (95% CI 0.16- 1.23; p < 0.087; I2 = 54.9%), as shown in Fig. 2.

**Table 1:** Baseline characteristics of patients with appendicular neoplasms and those without neoplasms. Association of appendiceal tumor in complicated and uncomplicated appendicitis

Author, year	Country	Study design	Appendicectomy			Age	Male	Female	CAP	UCAP
*Loftus *et al*.*, 2017	USA	Retrospective	677	Neoplasia	17	53 [35–61]	5 (29%)	12 (71%)	4 (23.50%)	13 (76.45%)
				Non- neoplasia	660	30 [22–45]	337 (51%)	323 (49%)	110 (16.67%)	550 (83.33%)
Brunner et al., 2019	Germany	Retrospective	1033	Neoplasia	29	57 [23–86]	17 (59%)	12 (41%)	15 (51.72%)	14 (48.28%)
				Non- neoplasia	1004	38 [18–94]	456 (45%)	548 (55%)	325 (32.37%)	679 (67.63%)
Bolmers et al., 2020	Netherlands	Retrospective	1941	Neoplasia	30	50.5 [25.8–70]	16 (53%)	14 (47%)	12 (40%)	18 (60%)
				Non- neoplasia	1911	29 [16–47]	1006 (53%)	905 (47%)	631 (33.02%)	1314 (66.98%)
Sugimoto et al.*,* 2022	Japan	Prospective	1277	Neoplasia	22	NR	NR	NR	14 (63.64%)	8 (36.36)
				Non- neoplasia	1255	NR	NR	NR	283 (22.55%)	972 (77.45%)
Total			4962	Neoplasia	98	54.8 [23–86]	38 (50%)	38 (50%)	45 (45.92%)	53 (54.08%)
				Non- neoplasia	4864	32.3 [16–94]	1799 (50.32%)	1776 (49.68%)	1349 (27.73%)	3515 (72.27%)

Abbreviation: CAP: complicated appendicitis; UCAP: uncomplicated appendicitis

The overall average age was 43.55 years, ranging from 16 to 94. Of the 3701 patients, 51% (n = 1887) were male, and 49% (n = 1814) were female. One study reported that those with complicated appendicitis and APNs were older than those with uncomplicated appendicitis and APNs (52.91 vs. 40.42, p =  < 0.001) (Table 2). No other studies compared the age of patients with APN in complicated and uncomplicated appendicitis.

**Table 2:** The incidence rate of APN in CAP vs. UCAP

Author, year	CAP	Neoplasia	UCAP	Neoplasia
Loftus et al., 2017	114	4	563	13
Brunner et al., 2019	340	15	693	14
Bolmers et al., 2020	643	12	1332	18
Sugimoto et al., 2022	297	14	980	8
Total	1394	45	3568	53

Abbreviation: CAP: complicated appendicitis; UCAP: uncomplicated appendicitis

### Secondary outcomes

#### Histology types

Seven studies reported on the histology of appendiceal neoplasms (Table 3). The most overall common appendiceal neoplasms were Neuroendocrine Tumors (NET) (49.21%), followed by Nonmucinous Adenocarcinoma (24.24%), Mixed Adeno-Neuroendocrine Tumor (MANEC) (11.40%), and Mucinous Adenocarcinoma (4.44%). Alajaaski et al. compared types of APNs in CAP versus UCAP in an overall cohort of 250 patients. In comparing CAP to UCAP, they found the following rates of APNs: adenocarcinoma (50% vs. 13%), NET (31% vs. 74%), and MANEC (19% vs. 13%) (p < 0.001) [8].

**Table 3:** Histopathology findings in appendiceal neoplasm

Author, year	Neoplasia	Benign	Malignant
Loftus et al., 2017	17	7 (4 carcinoids, 3 goblet cell carcinoids)	10 (5 adenocarcinomas, 2 mucinous adenocarcinomas, 1 signet ring adenocarcinoma, 1 adenosquamous carcinoma, 1 B-cell lymphoma)
Lietzén et al., 2018	462	65 Pseudomyxoma peritonei or mucinous	397 (232 NET, 52 MANEC, 113 Adenocarcinoma)
Brunner et al., 2019	29	13 (6 Adenoma, 6 LGMN, 1 HGMN)	16 (6 Adenocarcinoma, 9 NET, 1 Peritoneal carcinomatosis)
Westfall et al., 2019	23	1 globet cell carcinoid	22 (12 NET, 4 MANEC, 1 adenocarcinoma ex- globet cell, 1 signed ring cell, 2 mucinous adenocarcinomas, 1 mucinous neoplasm, 1 tubular carcinoid)
Bolmers et al., 2020	30	10 (adenoma)	20 (13 Grade 1 NET, 5 Goblet cell carcinoids, 1 Adenocarcinoma, 1 Mixed adeno-neuroendocrine carcinoma)
^*^Alajääski et al., 2022	**250**		**102 CAP (32 NET, 19 MANEC goblet cell, 51 Adenocarcinomas)** **148 UCAP (110 NET, 19 MANEC goblet cell, 19 Adenocarcinomas)**
Sugimoto et al., 2022	22	3 Adenoma, 5 LGMN	19 (6 Adenocarcinoma, 1 Cecal adenocarcinoma, 2 Metastatic cancer, 2 NET, 1 NEC, 2 Goblet cell adenocarcinoma)
Total	833	19 adenomas 35 Pseudomyxoma 11 LGMN 1 HGMN 4 carcinoids 4 globet cell carcinoids	410 NET 202 Adenocarcinoma 95 MANEC 37 Mucinous Adenocarcinoma 2 signet ring adenocarcinomas 1 adenosquamous carcinoma, 1 B-cell lymphoma

Abbreviation: CAP: complicated appendicitis; UCAP: uncomplicated appendicitis; Neuroendocrine tumor (NET); mixed adeno-neuroendocrine carcinomas (MANEC); Neuroendocrine carcinoma (NEC); low-grade mucinous neoplasm (LGMN), high-grade mucinous neoplasm (HGMN)

Alajääski *et al. *is the only study that differentiated the histology type between complicated and uncomplicated appendicitis

### GRADE approach to assess the auality of evidence

In evaluating the overall quality of evidence of two outcomes (incidence and histology), it was predominantly classified as low (n = 4) across all the comparisons examined (detailed data available in Supplementary eTable 2). The downgrading of evidence quality was primarily attributed to factors such as imprecision, indicated by minimal information size, very few events, and 95% confidence intervals overlapping with no significant effect. It is important to note that indirectness was not a concern in this analysis, as all the included studies directly compared interventions within the target populations of interest. Additionally, these studies measured the relevant outcomes necessary for patient evaluation. However, it is worth mentioning that inconsistencies were observed in the reporting of complicated and uncomplicated appendicitis and the histology type, which contributed to the seriousness of this issue within the available evidence.

## Discussion

Appendiceal tumors are relatively rare, with a reported incidence of approximately 1% in appendectomy specimens obtained from patients who have undergone the procedure for any reason. These tumors constitute a mere 0.5% of all gastrointestinal tumors [10]. Our meta-analysis reported an overall incidence of 1.98% of neoplasm in appendectomy specimens for appendicitis. The incidence rate of neoplasm in CAP was 3.29%, whereas in UCAP was 1.49%. This difference in incidence was not found to be statistically significant. CAP is defined as appendicitis with perforation and/or associated abscess. Conversely, UCAP is appendicitis without perforation or associated abscess. In clinical practice, A CT scan is an excellent modality to alert the surgeon whether the appendicitis is complicated or uncomplicated. Nevertheless, operative findings are more definitive in differentiating the severity and complexity of appendicitis.

Appendiceal neoplasm incidence has increased over time [11]. A retrospective study of 4765 patients, using the Surveillance, Epidemiology, and End Results database, reported an increased annual incidence of appendiceal neoplasm from 0.63% in 2000 to 0.97% in 2009 [12]. Our study showed an overall incidence of 1.98%. The reported increase in the incidence of ANs has yet to be attributed to a specific cause, and it remains uncertain if the mortality rate of ANs is following a similar trend. Additionally, it is unclear if the rise in frequency represents an actual change in the disease incidence or simply an increase in detection and reporting.

There is conflicting data about appendiceal neoplasm risk in CAP versus UCAP. A retrospective nationwide Finnish population-based registry study with 840 patients from 2007 to 2013 showed that the tumor risk was significantly higher in CAP compared with UCAP (3.24% vs. 0.87%, p < 0.001) [4]. Moreover, this study reported that neuroendocrine tumors were the most common neoplasm in UCAP. In contrast, adenocarcinoma was found to be common in CAP. However, Sugimoto et al., in a single-center retrospective study from 2013 to 2021, including 1277 patients, showed no difference in the rate of appendiceal neoplasm between CAP and UCAP (4.7% versus 1.4%, p = 0.7) [9]. It aligns with our study reporting a non-significant difference in 3.29% neoplasm rate in CAP and 1.49% in UCAP with an odds ratio of 0.44 (95% CI 0.16- 1.23; p < 0.087; I2 = 54.9%). Interestingly, Sugimoto et al. found that among patients aged ≥ 60 years, the incidence of appendiceal tumors was significantly higher in complicated than uncomplicated appendicitis (p = 0.006).

The World Health Organization (WHO) 2019 classified epithelial neoplasms of the appendix into two significant groups: neuroendocrine tumor (NET) and non-NET. NET tumors and adenocarcinomas are the top two most commonly occurring primary tumors of the appendix, accounting for 65% and 20% of overall cases, respectively [13]. Our results support this data, showing that the overall most common types of appendiceal neoplasms found were Neuroendocrine Tumors (NET) (49.21%), Nonmucinous Adenocarcinoma (24.24%), and Mixed Adeno-Neuroendocrine Tumor (MANEC) (11.40%). An England population-based analysis with 7056 incident cases of appendiceal tumors from 1995 to 2016 revealed an overall rising incidence of malignant neoplasms [14]. They postulated that changes to pathological classification systems have substantially impacted the rise in NET incidence rates and that the increase in adenocarcinomas may be due to environmental and genetic influences.

Previous studies have indicated a higher risk of appendiceal tumors with increasing age. A national database obtained from the Australian Institute of Health and Welfare (AIHW) from 1982 to 2013 showed that patients ≥ 50y age had a higher risk of having appendiceal neoplasm than patients < 50y age (IRR = 2.12, 95% CI: 1.89, 2.39, P value < 0.0001) [15]. Another retrospective review of 402 patients (36 patients with neoplasm) with CAP showed that patients with an appendiceal neoplasm were significantly older than those without neoplasm (56.6 years vs. 45.1 years, p < 0.01) [16]. The overall average age of patients with appendiceal neoplasm in our meta-analysis was 43.55 years, and those with complicated appendicitis and neoplasm were older than those with uncomplicated appendicitis and neoplasm (52.91 vs. 40.42, p =  < 0.001). This age difference may be partly due to the higher incidence of adenocarcinoma in complicated appendicitis, which tends to occur later in life compared to neuroendocrine tumors, which are more prevalent in patients with uncomplicated appendicitis.

Our present study has several strengths. To our knowledge, this is the first meta-analysis comparing rates and histology of appendiceal neoplasms in CAP versus UCAP. Given the large pooled data for a relatively uncommon disease (appendiceal neoplasm), this meta-analysis provides a more realistic incidence risk. The literature search was comprehensive, following a systematic methodology, applying pre-specified and detailed data tabulation and extraction and standardized evaluation of evidence quality and publication bias. Multiple researchers rigorously performed all steps. This approach facilitated the identification of a “clean” dataset from comparative studies of different methods to allow better generalizability of the results.

Our study has several limitations. We included retrospective and prospective comparative studies. Some eligible studies lacked data granularity on all characteristics or outcomes of interest; thus, the relative rates were estimated based on data availability.

Our study adds to the literature by breaking down rates and histology of neoplasm in CAP versus UCAP. Interestingly NET was more common in uncomplicated appendicitis, possibly due to its slow-growing and indolent nature, allowing the appendiceal lumen to accommodate growth rather than perforate. In contrast, Adenocarcinoma was more common in complicated appendicitis, likely due to this neoplasm's more aggressive, fast-growing, and penetrating nature. Our study encourages vigilance when evaluating a patient presenting with acute appendicitis. The practitioner must consider the severity of appendicitis (CAP vs UCAP) and the patient's age. We recommend that patients with CAP who were initially conservatively managed should strongly be considered for an interval appendectomy, especially if older. Additionally, we recommend further workup with colonoscopy and imaging before elective interval appendectomy in this patient population. Older patients with UCAP should also likely undergo an upfront appendectomy, given this population's rising and significant incidence of appendiceal neoplasm.

## Conclusion

This meta-analysis showed no difference in the incidence rate of appendiceal neoplasms between complicated and uncomplicated appendicitis. However, specific types of neoplasms differed between the two groups. Complicated appendicitis had a higher rate of adenocarcinoma, while uncomplicated appendicitis had higher rates of neuroendocrine tumors (NET). Mixed adeno-neuroendocrine tumors (MANEC) were observed in both groups. These findings emphasize the importance of evaluating risk factors for neoplasm when considering appendectomy in patients with appendicitis.

### Supplementary information

Below is the link to the electronic supplementary material.

Supplementary file1 (DOCX 26 KB)

## Data Availability

git@github.com:paosolis/Appendiceal-Neoplasm.git.
