# Supplementary material for: Incidence rate and histology of appendiceal neoplasms in complicated versus uncomplicated appendicitis: A meta-analysis and systematic review
Source: Langenbecks Arch Surg. 2023 Nov 9;408(1):432. doi: 10.1007/s00423-023-03164-0 (PMC10632310; doi:10.1007/s00423-023-03164-0)
Supplement: Supplementary file 1 — Supplementary file1 (DOCX 26 KB) [file 423_2023_3164_MOESM1_ESM.docx]

**Supplementary Online Content**

eMethods. Search protocol

eTable 1. Risk of Bias of Included Studies

eTable 2. GRADE Working Group grades of evidence (GradePro GDT)

**E methods: *SEARCH PROTOCOL***

A comprehensive search of several databases was conducted in English from inception to September 2022. The databases included Ovid MEDLINE(R) and Epub Ahead of Print, In-Process & Other Non-Indexed Citations, and Daily, Ovid EMBASE, Ovid Cochrane Central Register of Controlled Trials, Ovid Cochrane Database of Systematic Reviews, and Scopus. The search strategy was designed and conducted by an experienced librarian with input from the study's principal investigator. Controlled vocabulary supplemented with keywords was used to search for studies that compared the rates and types of appendiceal neoplasms between complicated and uncomplicated cases of appendicitis. The current strategy listing all search terms and how they are combined is available in the appendix.

**EMBASE**

[Appendix/ Or exp appendix perforation/ or exp appendix intussusception/ or exp appendix disease/ or processus vermiformis.mp. or vermiform process.mp. OR exp appendicitis/ or exp acute appendicitis/ OR

exp appendectomy or (appendix or appendices or appendi* or appendicitis or appendectomy).mp.

OR

(uncomplicate* or nonperforat* or non-perforat* or unperforat* or unruptur* or nonruptur* or non-ruptur* or (perforat* or rupture* or complicate*) or ((periappendicular or peri-appendicular or appendi*) adj3 (abscess or phlegmon or perforation))).mp.]

AND

exp appendix cancer/ or exp appendix tumor/ or exp appendix carcinoma/ OR exp Appendiceal Neoplasms/ or ((Appendi* or appendec*) adj3 (tumor* or turmour* or neoplasm* or carcinoid or carcinoma* or cancer* or malignan*)).mp. or pseudomyxoma.mp. or pseudomyxoma peritonei/

AND

exp risk factor/ Or exp incidence/ OR exp prevalence/ OR (risk or prevalence or incidence).ti,ab.

NOT ((exp animal/ or nonhuman/) NOT exp human/)

**MEDLINE OVID**

[exp Appendix/ or appendix.mp. or appendices.mp. or appendi*.mp. OR Appendicitis/ or appendicitis.mp. OR appendectomy.mp. or Appendectomy/

OR (uncomplicate* or nonperforat* or non-perforat* or unperforat* or unruptur* or nonruptur* or non-ruptur*).mp. OR

(perforat* or rupture* or complicate*).mp. OR ((periappendicular or peri-appendicular or appendi*) adj3 (abscess or phlegmon or perforation)).mp.]

AND

(exp Appendiceal Neoplasms/ or (tumor* or turmour* or neoplasm* or carcinoid or carcinoma* or cancer* or malignan*).mp.) adj3 (appendi* or appendec*).mp. OR Pseudomyxoma Peritonei/ or pseudomyxoma.mp.

AND

risk factors/ OR incidence/ or prevalence/ OR (risk or incidence or prevalence).ti,ab.

NOT

(Animals/ NOT (Animals/ AND Humans/))

**CINAHL**

( (MH "Appendix") OR "appendix" OR (MH "Appendicitis") OR (MH "Appendectomy") ) OR ( appendix OR appendices OR appendi* ) OR ( appendectomy or appendicitis )

OR

( uncomplicate* or nonperforat* or non-perforat* OR unruptur* ) OR ( non-ruptur* OR perforat* or rupture* OR complicate* ) OR ( (periappendicular OR peri-appendicular or appendi*) N3 (abscess or phlegmon or perforation)) )

AND

( "appendix cancer" OR appendiceal neoplasm* ) OR ( (appendi* or appendec*) N3 (tumor* or tumour* or neoplasm* or carcinoid* or carcinoma* OR cancer*or Malignan*) ) OR pseudomyxoma OR (MH "Pseudomyxoma Peritonei")

AND

(MH “Risk Factors”) OR (“incidence”) OR (MH “Prevalence”) or

TI ( risk or prevalence or incidence ) OR AB ( risk or prevalence or incidence )

**Cochrane**

#1 MeSH descriptor: [Appendix] explode all trees 45

#2 appendix or appendices or appendi* or appendicitis OR appendectomy 14186

#3 MeSH descriptor: [Appendicitis] explode all trees 653

#4 MeSH descriptor: [Appendectomy] explode all trees 537

#5 uncomplicate* or nonperforat* or non-perforat* or unperforat* or unruptur* or nonruptur* or non-ruptur OR perforat* or rupture* or complicate 23926

#6 (periappendicular or peri-appendicular or appendi*) NEAR/3 (abscess or phlegmon or perforation) 231

#7 MeSH descriptor: [Appendiceal Neoplasms] explode all trees 13

#8 MeSH descriptor: [Pseudomyxoma Peritonei] explode all trees 6

#9 ((appendi* or appendec*) NEAR/3 (tumor* or turmour* or neoplasm* or carcinoid or carcinoma* or cancer* or malignan*)) OR pseudomyxoma 378

#10 MeSH descriptor: [Risk Factors] explode all trees 26361

#11 MeSH descriptor: [Incidence] explode all trees 10776

#12 MeSH descriptor: [Prevalence] explode all trees 4979

#13 risk or incidence or prevalence 388327

#14 #1 OR #2 OR #3 OR #4 OR #5 OR #6 35710

#15 #7 OR #8 OR #9 378

#16 #10 OR #11 OR #12 OR #13 388327

#17 #14 AND #15 AND #16 250

**SCOPUS**

( TITLE-ABS-KEY ( appendiceal AND neoplasms ) OR TITLE-ABS-KEY ( ( appendi* OR appendec* ) W/3 ( tumor* OR turmour* OR neoplasm* OR carcinoid OR carcinoma* OR cancer* OR malignan* ) )OR TITLE-ABS-KEY(Pseudomyxoma Peritonei OR pseudomyxoma) OR TITLE-ABS-KEY ( ( periappendicular OR peri-appendicular OR appendi* ) W/3 ( abscess OR phlegmon OR perforation ) ) )

AND

( ( TITLE-ABS-KEY ( ( appendix OR appendices OR appendi* OR appendicitis OR appendectomy ) ) ) Or ( TITLE-ABS-KEY ( uncomplicate* OR nonperforat* OR non-perforat* OR unperforat* OR unruptur* OR nonruptur* OR non-ruptur* ) OR TITLE-ABS-KEY ( perforat* OR rupture* OR complicate* ) OR TITLE-ABS-KEY ( ( periappendicular OR peri-appendicular OR appendi* ) W/3 ( abscess OR phlegmon OR perforation ) ) ) ) ) AND NOT ( TITLE-ABS-KEY ( ( animal* ) ) )

**Web of Science - searched Science Citation Index (SCI), Conference Proceedings Citation Index (CPCI) and BIOSIS Citation Index (BCI)**

All databases-Web of Science Core Collection, Biological Abstracts, Biosis, KCI- Korean journal Database, Medline, Scielo Citation Index

TS=(appendix or appendices or appendi* or appendicitis or appendectomy) OR TS=(uncomplicate* or nonperforat* or non-perforat* or unperforat* or unruptur* or nonruptur* or non-ruptur*) OR TS=(perforat* or rupture* or complicate*) OR TS=((periappendicular or peri-appendicular or appendi*) NEAR/3 (abscess or phlegmon or perforation))

AND

TS=( Appendiceal Neoplasms) or TS= ((appendi* or appendec*) NEAR/3 (tumor* or turmour* or neoplasm* or carcinoid or carcinoma* or cancer* or malignan*)) OR TS=(Pseudomyxoma Peritonei or pseudomyxoma)

AND

TS=(risk or incidence or prevalence)

NOT

TS=(animal*)

| eTable 1. Risk of Bias of Included Studies | | | | | | | | |  |
| --- | --- | --- | --- | --- | --- | --- | --- | --- | --- |
| Study | Score | | | | | | | |  |
| CLARITY Tool to Assess Risk of Bias in Cohort Studies | 1. Was the selection of exposed and non-exposed cohorts drawn from the same population? | 2. Can we be confident in the assessment of exposure? | 3. Can we be confident that the outcome of interest was not present at the start of the study? | 4. Did the study match exposed and unexposed for all variables that are associated with the outcome of interest, or did the statistical analysis adjust for these prognostic variables? | 5. Can we be confident in the assessment of the presence or absence of prognostic factors? | 6. Can we be confident in the assessment of the outcome? | 7. Was the follow-up of cohorts adequate? | 8. Were co-interventions similar between groups? | The overall risk of bias |
| *Loftus et al*, 2017 | Low | Low | Low | Low | Low | Low | Low | Low | Low |
| *Lietzén et al., 2018* | Low | Low | Low | Low | Moderate | Moderate | Low | Low | Moderate |
| Brunner et al., 2019 | Low | Low | Low | Low | Low | Low | Low | Low | Low |
| Westfall et al., 2019 | Low | Low | Low | Low | Low | Low | Low | Low | Low |
| Bolmers et al., 2020 | Low | Low | Low | Low | Low | Moderate | Low | Low | Moderate |
| Alajääski et al., 2022 | Low | Low | Low | Low | Low | Moderate | Moderate | Low | Moderate |
| Sugimoto et al., 2022 | Low | Low | Low | Low | Low | Low | Low | Low | Low |

**eTable 2. GRADE Working Group grades of evidence.**

**Bibliography:** Schünemann, H. et al. (eds) (2013) GRADE handbook for grading the quality of evidence and strength of recommendations, The GRADE Working Group. Available at: https://gdt.gradepro.org/app/handbook/handbook.html#h.ged5uqebmir9 (Accessed: 2022).

| **Certainty assessment** | | | | | | | **№ of patients** | | **Effect** | | **Certainty** | **Importance** |
| --- | --- | --- | --- | --- | --- | --- | --- | --- | --- | --- | --- | --- |
| **№ of studies** | **Study design** | **Risk of bias** | **Inconsistency** | **Indirectness** | **Imprecision** | **Other considerations** | **Complicated appendicitis** | **uncomplicated appendicitis** | **Relative (95% CI)** | **Absolute (95% CI)** |  |  |
| **Incidence rate** | | | | | | | | | | | | |
| 4 | observational studies | not serious | not serious | not serious | not serious | none | 45/1394 (3.2%) | 53/3568 (1.5%) | **OR 0.44** (0.16 to 1.23) | **8 fewer per 1,000** (from 12 fewer to 3 more) | ⨁⨁◯◯ Low | CRITICAL |
| **Histology (assessed with pathology report)** | | | | | | | | | | | | |
| 4 | observational studies | not serious | not serious | not serious | not serious | none |  | | | | ⨁⨁◯◯ Low | IMPORTANT |

**CI:** confidence interval; **MD:** mean difference; **OR:** odds ratio

High certainty: we are confident that the true effect is close to the effect estimates.

Moderate certainty: we are moderately confident in the effect estimate; the true effect is likely to be close to the estimate of the effect, but there is a possibility that it is substantially different.

Low certainty: our confidence in the effect estimate is limited; the true effect may be substantially different from the estimate of the effect.

Very low certainty: we have very little confidence in the effect estimate; the true effect is likely to be substantially different from the estimate of the effect.

**Explanations**

a. I^2 = 85.7 %

b. The overall risk of bias to Dolejs et al., 2017 is High, and to Mlambo et al., 2022 is unclear

c. The sample size is not very large (at least 2000 and perhaps 4000 patients)

d. I^2 = 86.6 %

e. I^2 = 89.5%
